# 12-O-Tetradecanoylphorbol-13-acetate (TPA) is anti-tumorigenic in liver cancer cells via inhibiting YAP through AMOT

**DOI:** 10.1038/srep44940

**Published:** 2017-03-21

**Authors:** Guoqing Zhu, Yan Chen, Xiao Zhang, Qi Wu, Yinghui Zhao, Yuxin Chen, Fenyong Sun, Yongxia Qiao, Jiayi Wang

**Affiliations:** 1 Department of Clinical Laboratory, Shanghai Tenth People’s Hospital of Tongji University, Shanghai, 200072, China; 2 School of Public Health, Shanghai Jiaotong University School of Medicine, Shanghai, 200025, China; 3 Tongji University Advanced Institute of Translational Medicine, Shanghai, 200092, China

## Abstract

TPA stimulates carcinogenesis in various types of cancers. However, we found that TPA inhibits transformative phenotypes in liver cancer cells via the translocation of YAP from the nucleus, where it functions as a transcriptional co-factor, to the cytoplasm. Such effects led to a separation of YAP from its dependent transcription factors. The inhibitory effects of TPA on YAP were AMOT dependent. Without AMOT, TPA was unable to alter YAP activity. Importantly, the depletion of YAP and AMOT blocked the TPA-reduced transformative phenotypes. In sum, TPA has been established as an anti-tumorigenic drug in liver cancer cells via YAP and AMOT.

TPA, also called phorbol 12-myristate 13-acetate (PMA), is a small molecule drug that activates the signal transduction enzyme protein kinase C (PKC) by directly binding to its C1 domains^1^,^2^. Commonly, TPA is employed as a tumor-promoting agent for skin carcinogenesis in rodents and is associated with increased cell proliferation in malignant cells from several types of tumors, such as melanoma and breast and oral cancer^3^,^4^,^5^. However, the function of TPA is controversial because a decrease in cell proliferation capacity has also been observed in TPA-treated lymphoma cells compared to controls^6^,^7^. Hence, different effects may occur after exposure to TPA in different types of cells. Although some studies have investigated the effects of TPA on cell proliferation in liver cancer^8^,^9^, the exact roles of TPA in maintaining transformative phenotypes in liver cancer cells remain largely unknown.

Liver cancer is the fifth most common cancer type globally and the third most frequent cause of cancer–related mortality worldwide^10^. Liver cancer carries a poor prognosis because the treatment options are extremely limited. Curative resection or transplantation is currently the best curative option for treatment, yet recurrence and metastasis are quite common in patients^11^. Recently, transcatheter arterial chemoembolization (TACE) has been used for patients who cannot receive these local eradication methods due to reasons such as poor residual liver function, complicated tumor location, or complications^12^. However, TACE may have negative effects on liver function; therefore, it is urgent to improve the therapeutic strategies^13^, and the development of potential drugs might be a promising option.

Recently, emerging evidence has shown the critical roles of the tumor suppressor Hippo signaling pathway in the pathogenesis of various cancers, including liver cancer^14^,^15^. Yes-associated protein (YAP), the major downstream effector of this pathway, has been identified as an oncoprotein that is also critical for the initiation and progression of liver cancer^16^. YAP is phosphorylated and inhibited by Hippo signaling, thereby resulting in its translocation from the nucleus into the cytoplasm, where its activity is lost^17^. In the nucleus, the activity of YAP largely depends on its interaction with its dependent transcription factors, such as the TEAD family, Runx2, CREB, and p73 proteins^17^,^18^,^19^,^20^.

Angiomotin (AMOT) contains conserved glutamine-rich domains and PPxY motifs in its N-terminus, through which it binds to a number of WW domain-containing proteins^21^. Interestingly, YAP contains WW domains^22^. Some studies have suggested that AMOT can interact with YAP to inhibit the growth of liver and breast cancer cells^23^, indicating that AMOT may play an essential suppressive role to tumorigenesis. AMOT also promotes YAP phosphorylation through activating the LATS kinase, subsequently transferring YAP from the nucleus to the cytoplasm^24^. Moreover, AMOT may compete with PPxY motif-containing transcription factors for YAP binding, for example, inhibiting YAP-TEAD binding to decrease the transcription of TEAD-target genes^25^.

Here, we intended to investigate the function of TPA in liver cancer cells. We have also investigated the underlying mechanism of how TPA exerts its roles in liver cancer cells. This study might provide valuable information for improving liver cancer treatments in the future.

## Materials and Methods

The methods were carried out in accordance with approved guidelines, and the experimental protocols were approved by the Department of Clinical Laboratory, Shanghai Tenth People’s Hospital of Tongji University (Shanghai, 200072, China).

### Cell culture and vectors

The liver cancer cell lines Bel-7402 and Bel-7404 were cultured in DMEM. Cells were treated with TPA (final concentration 16–48 μM, Beyotime, Haimen, China) for 24 h before harvest for further analysis. The TEAD-Gal4/pUAS-LUC, HULC-promoter, YAP-FLAG, TEAD4-Myc, CREB-HA, Runx2-HA, AMOT-HA, YAP-sh1 and –sh2, and AMOT-sh1 and –sh2 were obtained from our previous studies^26^,^27^,^28^. ShRNAs specifically targeting TAZ (TAZ-sh1 and –sh2) were purchased from Biolink LTD (Shanghai, China).

### Immunofluorescence (IF) and Western Blotting (WB)

For IF, the primary antibodies used were anti-YAP (Abcam, Hong Kong, China, #ab52771), anti-TEAD4 (Epitomics, Burlingame, CA, USA, #s1666), anti-CREB (Epitomics, #1496), anti-p73 (Epitomics, #1636), anti-Runx2 (Epitomics, #5356), anti-HA (Cell Signaling Technology (CST), Cambridge, MA, USA, #3724) or anti-AMOT (Abcam, #ab85143). The slides were incubated with Alexa Fluor^®^-488/555 fluorescently conjugated secondary antibodies (CST, #4408, #4412, #4409 or #4413) for 1 h in the dark before being mounted with ProLong^®^ Gold antifade reagent with DAPI (Molecular Probes, Eugene, OR, USA). The sides were then observed using an LSM 800 Confocal Microscope (Carl Zeiss AG, Oberkochen, Germany).

For WB, the primary antibodies used were anti-p-YAP (Ser127) (CST, #13619), anti-YAP (Abcam, #ab52771), anti-GAPDH (CST, #5176), anti-β-tubulin (CST, #2128), anti-Histone-H3 (Santa Cruz Biotechnology, Santa Cruz, CA, USA, #sc-10809), anti-CREB (Epitomics, #1496), anti-Runx2 (Epitomics, #5356), anti-TEAD4 (Epitomics, #s1666), anti-p73 (Epitomics, #1636), anti-TAZ (CST, #2149), or anti-AMOT (Abcam, #ab85143). For cytosolic and nuclear fractionation, a nuclear extraction kit from Active Motif (Carlsbad, CA, USA) was used. The membranes were incubated with secondary antibodies conjugated with horseradish peroxidase (CST, #7074 or #7076) and visualized using Pierce ECL Western Blotting Substrate (Thermo Fisher Scientific, Waltham, MA, USA). The software ImageJ version 1.47, (Bethesda, MD, USA) was used for densitometry of the Western blots.

All IF and WB were performed by conventional methods, and the protocols are available elsewhere.

### Cell proliferation, Caspase3/7 activity, soft-agar colony formation assay and quantitative RT-PCR (qPCR)

Cell proliferation, Caspase3/7 activity, soft agar colony formation and quantitative RT-PCR (qPCR) assays were performed as described previously^29^. The primers used for qPCR are listed below: CTGF-qPCR-F: 5′ CCTGTGCAGCATGGACGTTCGT 3′, CTGF-qPCR-R: 5′ AACGTGTCTTCCAGTCGGTAAG 3′; ANKRD1-qPCR-F: 5′ GAAACAACGAGAGGCAGAGCTC 3′, ANKRD1-qPCR-R: 5′ AGAAACGTAGGCACATCCACAG 3′; MCAM-qPCR-F: 5′ GCGTCTACAAAGCTCCGGAGGA 3′, MCAM-qPCR-R: 5′ GAATGTGGACCCGGTTCTTCTCCTC 3′; HULC-qPCR-F: 5′ ACCTCCAGAACTGTGATCCAAAATG 3′, HULC-qPCR-R: 5′ CAAATTTGCCACAGGTTGAACAC 3′ and GAPDH-qPCR-F: 5′ ATCATCCCTGCCTCTACTGG 3′, GAPDH-qPCR-R: 5′ GTCAGGTCCACCACTGACAC 3′.

### Co-immunoprecipitation (co-IP)

Co-IP was performed as described previously^30^. The reagents used were protein A/G-Sepharose (Novex, Oslo, Norway) and Western/IP lysis buffer (Beyotime, Haimen, China). The antibodies used for IP were anti-YAP (Santa Cruz Biotechnology, #sc101199) or anti-FLAG (CST, #8146).

### Mice experiments

Bel-7402 cells (5 × 10^6 cells) were subcutaneously injected into 8-week-old athymic nude mice (Bikai, Shanghai, China). After xenografts were visible (10^th^ day after injection), the mice were treated with TPA (100 μg/kg) for another 20 days before the tumor sizes were measured. The tumor volume was calculated as 0.5 × L × W^2, where L is length and W is width. All mouse experiments were performed according to the institutional guidelines of the Shanghai Tenth People’s Hospital.

### Statistical analysis

Tests to examine the differences between groups included Student’s t test and one-way ANOVA; p < 0.05 was regarded as statistically significant.

## Results

### TPA inhibits transformative phenotypes and YAP activity in liver cancer cells

**Figure 1:**
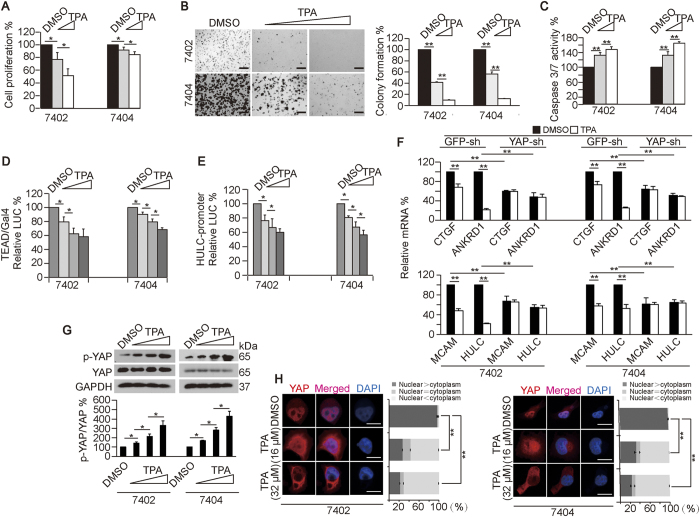
TPA inhibits transformative phenotypes and YAP in liver cancer cells. (**A**–**C**) TPA dose-dependently (final concentration 16–32 μM) decreased cell proliferation and colony formation capacities while increasing apoptosis, as measured by an MTT assay (**A**), a soft agar colony formation assay (**B**) and the Caspase 3/7 luciferase Glo reagent (**C**), respectively. Scale bar, 500 μm. (**D**,**E**) TPA (final concentration 16–48 μM) reduced the YAP-dependent transcription factor activity of TEAD and CREB, as measured using a TEAD-Gal4/pUAS-LUC (for testing TEAD activity) and HULC-promoter reporter (for testing CREB activity). (**F**) TPA reduced TEAD and CREB target gene expression. mRNA levels of TEAD target genes (CTGF and ANKRD1) and CREB target genes (MCAM and HULC) in control cells and Bel-7402 or Bel-7404 cells with YAP knocked down in the presence or absence of DMSO or TPA (final concentration 16 μM). (**G**) Representative Western blots of p-YAP and YAP in Bel-7402 and Bel-7404 cells treated with DMSO or increasing concentrations of TPA (final concentration 16–48 μM) (upper panel). The p-YAP levels were normalized to the total-YAP levels, and the data are graphed in the lower panel. (**H**) TPA (final concentration 16–32 μM) translocated YAP from the nucleus to the cytoplasm. The subcellular localization of YAP was analyzed by IF using anti-YAP antibodies. Scale bar, 20 μm. The bar graphs are shown as the percent of cells in each of three categories (nuclear > cytoplasm, nuclear = cytoplasm, and nuclear < cytoplasm) from 100 randomly counted cells. The data are shown as the mean ± SD from three independent experiments. The data from cells treated with DMSO infected with or without GFP-sh are arbitrarily set to 100% (except Fig. 1H). *p < 0.05 and **p < 0.01, as analyzed using one-way ANOVA. Specifically in Fig 1H, comparisons of the percent of the cells categorized into the “nuclear > cytoplasm” group among different treatments, as indicated, are also statistically analyzed.

We found that the cell-proliferation and colony-formation capacities of Bel-7402 and Bel-7404 cells could be dose-dependently inhibited by increasing concentrations of TPA (Fig. 1A,B). By contrast, Caspase 3/7 activities could be dose-dependently induced (Fig. 1C). These results suggested that TPA inhibits the transformative phenotypes of liver cancer cells.

Because YAP is a transcription co-factor^17^ and its activity relies on its dependent transcription factors (including TEAD and CREB)^31^,^32^, we tested whether TPA treatment influences the transcriptional activities of TEAD and CREB. We found that YAP-dependent TEAD and CREB activities could be dose-dependently reduced by TPA, as measured using a TEAD-Gal4/pUAS-LUC and an HULC-promoter luciferase reporter, which contains a CREB-responses element, before and after treating cells with increasing concentrations of TPA (Fig. 1D,E). TEAD target genes, CTGF and ANKRD1^33^,^34^, and CREB target genes, MCAM and HULC^35^,^36^, were also found to be downregulated by TPA treatment (Fig. 1F). Moreover, the inhibitory efficacies of TPA on the mRNA levels of these genes were abolished when YAP was depleted (Fig. 1F), further demonstrating that TPA can inhibit the activities of YAP-dependent transcription factors.

Then, we investigated whether TPA directly affected the phosphorylation of YAP (p-YAP), a hallmark of YAP inactivation^37^. We found that p-YAP was dose-dependently elevated, while total YAP levels were unaffected by increasing concentrations of TPA (Fig. 1G). Further, we found that TPA was able to shuttle YAP from the nucleus, where YAP exerts its major function on tumorigenesis^38^, to the cytoplasm (Fig. 1H); these effects were also dose-dependent, suggesting YAP can be directly inhibited by TPA.

### TPA separates YAP from its dependent transcription factors

**Figure 2:**
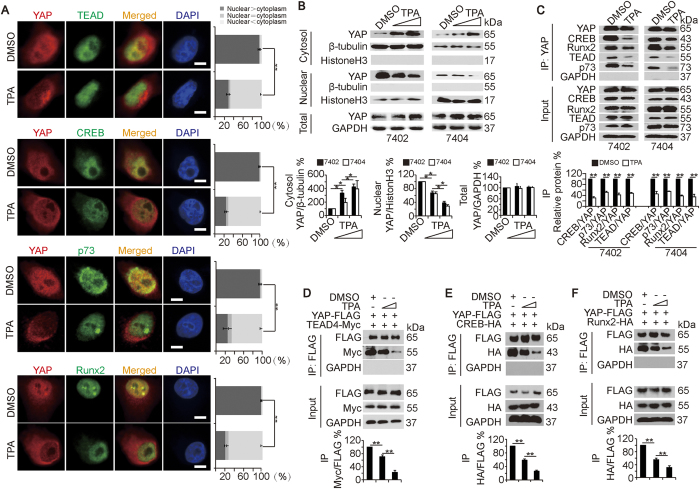
TPA separates YAP from its dependent transcription factors. (**A**) TPA (final concentration 16 μM) separated YAP from TEAD, CREB, p73 and Runx2, as measured by IF using the indicated antibodies in Bel-7402 cells. Scale bar, 20 μm. The bar graphs are shown as the percent of cells in each of three categories (nuclear > cytoplasm, nuclear = cytoplasm, and nuclear < cytoplasm) from 100 randomly counted cells. (**B**) TPA induced cytosolic expression of YAP while reducing the nuclear expression of YAP. Cytosolic and nuclear fractionation analysis was performed in Bel-7402 and Bel-7404 cells treated with DMSO or increasing concentrations of TPA (final concentration 16–32 μM). The representative WB images from three independent experiments are shown in the upper panel, and the relative ratio are shown in the lower panel. The data from the “DMSO” group are arbitrarily set to 100%. (**C**) TPA (final concentration 16 μM) inhibited the interaction between YAP and its dependent transcription factors. YAP was immuno-precipitated by anti-YAP antibodies, and co-immuno-precipitations of CREB, Runx2, TEAD, and p73 were measured by WB. The representative WB images from three independent experiments are shown in the upper panel. The relative ratios are shown in the lower panel. The data from the “DMSO” group are arbitrarily set to 100%. (**D**–**F**) TPA (final concentration 16–32 μM) dose-dependently dissociated exogenous YAP-FLAG from TEAD4-Myc (**D**), CREB-HA (**E**), or Runx2-HA (**F**). Exogenous YAP-FLAG was immuno-precipitated by anti-FLAG antibodies, and co-immuno-precipitations of TEAD4-Myc, CREB-HA and Runx2-HA were measured by WB using the indicated antibodies. The representative WB images from three independent experiments are shown in the upper panel. The relative ratios are shown in the lower panel. The data from the “DMSO” group are arbitrarily set to 100%. The data are shown as the mean ± SD from three independent experiments. *p < 0.05 and **p < 0.01, as analyzed using Student’s t test (Fig. 2A and C) and one-way ANOVA (Fig. 2B and D–F). Specifically in Fig. 2A, comparisons of the percent of the cells categorized into the “nuclear > cytoplasm” group between different treatments, as indicated, are also statistically analyzed.

Because YAP activity relies on its transcription factors^16^, we performed IF and found that TPA treatments led to YAP translocation from the nucleus to the cytoplasm (Fig. 2A). However, the nuclear localization of YAP-dependent transcription factors, including TEAD, CREB, p73 and Runx2, was not altered in Bel-7402 cells (Fig. 2A). Similarly, data from fractionation experiments indicated that TPA treatments led to gradually increasing cytoplasmic accumulation of YAP, whereas decreased nuclear expression of YAP was caused by increasing concentrations of TPA in both Bel-7402 and Bel-7404 cells (Fig. 2B). Further, co-IP experiments demonstrated that TPA treatments dissociated endogenous YAP from endogenous CREB, Runx2, TEAD and p73 in Bel-7402 and Bel-7404 cells (Fig. 2C). In Bel-7402 cells co-transfected with exogenous YAP-FLAG and TEAD4-Myc, CREB-HA, or Runx2-HA, we also found that TPA treatments inhibited the interactions between YAP-FLAG and its dependent exogenous transcription factors (Fig. 2D,F). Taken together, TPA separates YAP from its dependent transcription factors.

### AMOT overexpression has similar effects as TPA

**Figure 3:**
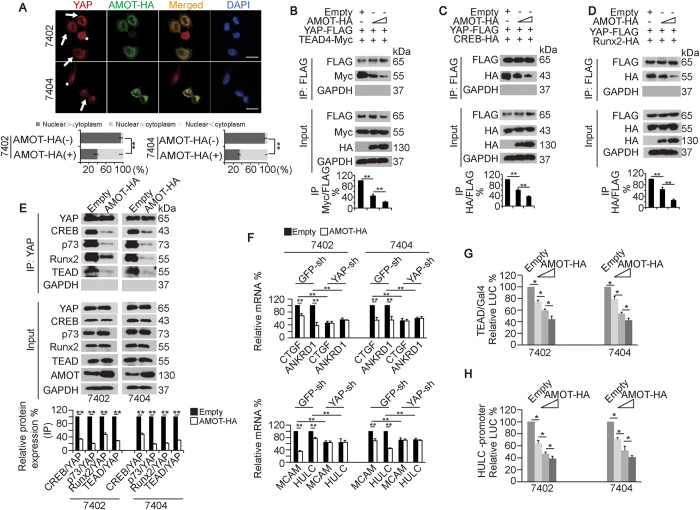
Overexpression of AMOT inhibits YAP activity. (**A**) AMOT drove YAP from nucleus to cytoplasm, as measured by IF. The arrows indicate cells successfully transfected with exogenous AMOT-HA, while the asterisks indicate cells without successful transfection. Scale bar, 50 μm. The bar graphs are shown as the percent of cells in each of three categories (nuclear > cytoplasm, nuclear = cytoplasm, and nuclear < cytoplasm) from 100 randomly counted cells with ( + ) or without (–) transfection of AMOT-HA. (**B**–**D**) Overexpression of AMOT-HA inhibited the interaction between YAP-FLAG and its dependent transcription factors. YAP-FLAG and TEAD4-Myc (**B**), CREB-HA (**C**) or Runx2-HA (**D**) co-transfected Bel-7402 cells were transfected with increasing concentrations of AMOT-HA. Exogenous YAP-FLAG was immuno-precipitated by anti-FLAG antibodies, and co-immuno-precipitations of TEAD4-Myc, CREB-HA or Runx2-HA were measured by WB using the indicated antibodies. Representative WB images from three independent experiments are shown in the upper panel. The relative ratioss are shown in the lower panel. (**E**) Overexpression of AMOT suppressed the interaction of endogenous YAP with its dependent transcription factors. Endogenous YAP was immuno-precipitated by anti-YAP antibodies, and co-immuno-precipitations of CREB, p73, Runx2 and TEAD were measured by WB using the indicated antibodies. Representative WB images from three independent experiments are shown in the upper panel. The relative ratios are shown in the lower panel. (**F**) mRNA levels of CTGF, ANKRD1, MCAM and HULC were measured in Bel-7402 and Bel-7404 cells with or without YAP knockdown in the presence or absence of AMOT-HA overexpression. (**G**–**H**) TEAD and CREB-dependent transcription activities were measured in control cells and Bel-7402 or Bel-7404 cells transfected with increasing concentrations of AMOT-HA by a TEAD-Gal4/pUAS-LUC and HULC-promoter reporter luciferase system, respectively. All the data from cells transfected with Empty vectors are arbitrarily set to 100%. The data are shown as the mean ± SD from three independent experiments. *p < 0.05 and **p < 0.01. The data in Fig. 3A and E were analyzed using Student’s t-test and the data in Fig. 3B–D and F–H was analyzed using one-way ANOVA. Specifically in Fig. 3A, comparisons of the percent of the cells categorized into the “nuclear > cytoplasm” group between indicated treatments are also statistically analyzed.

We have previously reported that AMOT overexpression causes the inhibition of YAP^28^. Here, cytoplasmic accumulation of YAP could be found in Bel-7402 and Bel-7404 cells successfully transfected with exogenous AMOT-HA, whereas nuclear accumulation of YAP was observed in the cells without successful transfection (Fig. 3A), demonstrating that AMOT overexpression can drive YAP from the nucleus to the cytoplasm. We also found that AMOT overexpression dose-dependently suppressed the interactions between exogenous YAP-FLAG and TEAD4-Myc, CREB-HA or Runx2-HA (Fig. 3B–D). Further, the amounts of CREB, p73, Runx2 and TEAD in the immunoprecipitates that were pulled down by anti-YAP antibodies were greatly reduced when AMOT was overexpressed (Fig. 3E). Moreover, AMOT overexpression reduced the mRNA levels of CTGF, ANKRD1, MCAM and HULC; however, the depletion of YAP blocked such effects (Fig. 3F), suggesting that AMOT controls TEAD and CREB target gene transcription, possibly via YAP. Furthermore, the transcriptional activities of TEAD and CREB measured by luciferase-based experiments were dose-dependently reduced in Bel-7402 and Bel-7404 cells transfected with increasing concentrations of exogenous AMOT-HA compared to those from the control cells (Fig. 3G,H). These results suggested that, similar to the effects of TPA, AMOT overexpression is capable of inhibiting YAP-dependent transcriptional activity.

### TPA simultaneously induces the translocation of both YAP and AMOT from the nucleus to the cytoplasm

**Figure 4:**
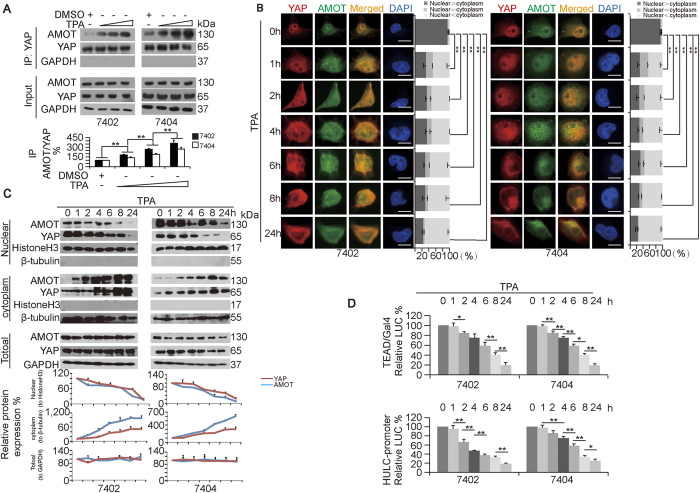
TPA simultaneously translocates YAP and AMOT from the nucleus to the cytoplasm. (**A**) TPA enhanced the interaction between YAP and AMOT. Bel-7402 and Bel-7404 cells were treated with DMSO or increasing concentrations of TPA (final concentration 16–48 μM); endogenous YAP was immuno-precipitated by anti-YAP, and the co-immuno-precipitation of AMOT was measured by anti-AMOT antibodies. Representative WB images from three independent experiments are shown in the upper panel. The relative ratios of AMOT to YAP in the IP samples are shown in the lower panel. The data from the “DMSO” group are arbitrarily set to 100%. (**B**) Subcellular localization of YAP and AMOT in Bel-7402 and Bel-7404 cells treated with TPA (final concentration 16 μM) at the indicated time points. The bar graphs are shown as the percent of cells in each of three categories (nuclear > cytoplasm, nuclear = cytoplasm, and nuclear < cytoplasm) from 100 randomly counted cells. (**C**) Cytosolic and nuclear fractionation experiments in Bel-7402 and Bel-7404 cells treated with TPA (final concentration 16 μM) for the indicated time. Representative WB images from three independent experiments are shown in the upper panel. The indicated relative ratios of AMOT or YAP to Histone-H3, β-tubulin or GAPDH are shown in the lower panel. The data from the “0 h” group are arbitrarily set to 100%. (**D**) TPA reduced the luciferase activities of the TEAD-Gal4/pUAS-LUC and HULC-promoter reporter in Bel-7402 and Bel-7404 cells treated with TPA (final concentration 16 μM) for the indicated times. The data from the “0 h” group are arbitrarily set to 100%. The data are shown as the mean ± SD from three independent experiments. *p < 0.05 and **p < 0.01. The data from Fig. 4A,B and D were analyzed using one-way ANOVA. Specifically in Fig. 4B, comparisons of the percent of the cells categorized into the “nuclear > cytoplasm” group among different treatments, as indicated, are also statistically analyzed.

Next, we tested whether the interaction between AMOT and YAP can be affected by TPA. We found that TPA dose-dependently enhanced the AMOT-YAP interaction in both Bel-7402 and Bel-7404 cells (Fig. 4A). IF experiments revealed the gradual and simultaneous translocation of both YAP and AMOT from the nucleus to the cytoplasm (Fig. 4B). By cytosolic and nuclear fractionation experiments, the gradual and simultaneous translocation of YAP and AMOT from nucleus to the cytoplasm in both Bel-7402 and Bel-7404 cells was also observed (Fig. 4C). These results suggested that the changes in the subcellular localization of YAP and AMOT are similar. Further, luciferase-based experiments demonstrated that the reduction in the transcriptional activities of TEAD and CREB occurred almost simultaneously with the subcellular alterations of AMOT and YAP in both Bel-7402 and Bel-7404 cells (Fig. 4D).

### AMOT is essential for the TPA-induced inhibition of YAP

**Figure 5:**
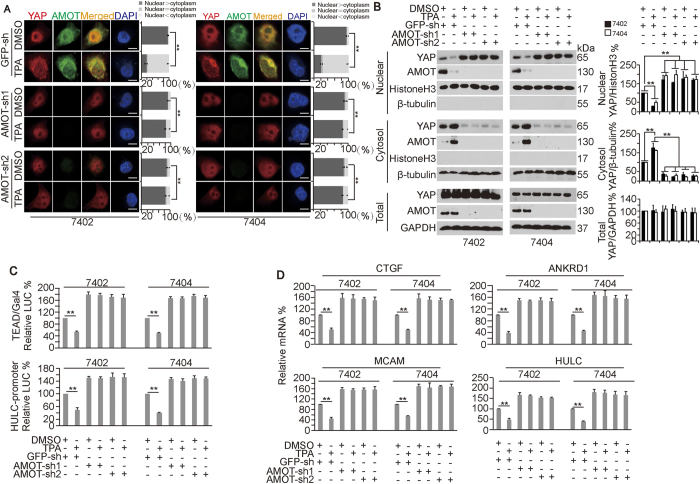
AMOT is essential for the TPA-dependent inhibition of YAP activity. (**A**) Subcellular localization of YAP and AMOT in control cells and Bel-7402 or Bel-7404 cells with AMOT knocked down (infected with AMOT-sh1 and –sh2, respectively) in the presence or absence of DMSO or TPA (final concentration 16 μM). The bar graphs are shown as the percent of cells in each of three categories (nuclear > cytoplasm, nuclear = cytoplasm, and nuclear < cytoplasm) from 100 randomly counted cells. (**B**) Cytosolic and nuclear fractionation experiments on control cells and Bel-7402 or Bel-7404 cells with AMOT knocked down in the presence or absence of DMSO or TPA (final concentration 16 μM). Representative WB images from three independent experiments are shown in the left panel, and the relative ratios between nuclear-YAP and nuclear-Histone H3, between cytosolic-YAP and cytosolic-β-tubulin, and between total-YAP and total-GAPDH are shown in the right panel. (**C**) Luciferase activities from the TEAD-Gal4/pUAS-LUC and HULC promoter reporter system in control cells and Bel-7402 or Bel-7404 cells with AMOT knocked down in the presence or absence of treatment with DMSO or TPA (final concentration 16 μM). (**D**) mRNA levels of CTGF, ANKRD1, MCAM and HULC in control cells and Bel-7402 or Bel-7404 cells with AMOT knocked down in the presence or absence of DMSO or TPA (final concentration 16 μM). The data are shown as the mean ± SD from three independent experiments. The data from cells infected with GFP-sh and treated with DMSO are arbitrarily set to 100% (except Fig. 5A). **p < 0.01. The data from Fig. 5A and B–D were analyzed using Student’s t-test and one-way ANOVA, respectively. Specifically in Fig 5A, comparisons of the percent of the cells categorized into the “nuclear > cytoplasm” group between different treatments, as indicated, are also statistically analyzed.

Next, we sought to determine whether AMOT is essential for the TPA-induced inhibition of YAP. We found that YAP translocation from the nucleus to the cytoplasm was blocked when AMOT was knocked down by two independent shRNAs against AMOT compared to the control in both Bel-7402 and Bel-7404 cells (Fig. 5A).

Further, cytosolic and nuclear fractionation experiments demonstrated that the knockdown of AMOT could only increase the nuclear expression of YAP while decreasing the cytoplasmic expression of YAP, but AMOT knockdown blocked the TPA-induced translocation of YAP from the nucleus to the cytoplasm in both Bel-7402 and Bel-7404 cells (Fig. 5B). Moreover, the TPA-induced reduction of TEAD and CREB transcriptional activities was abolished when AMOT was knocked down (Fig. 5C). Further, the TPA-reduced downregulation of the mRNA levels of CTGF, ANKRD1, MCAM and HULC in the control cells could not be observed in Bel-7402 and Bel-7404 cells with AMOT knocked down (Fig. 5D). These results demonstrated that the TPA-induced inhibitory effects on YAP rely on the function of AMOT.

### TPA-reduced transformative phenotypes rely on YAP and AMOT

**Figure 6:**
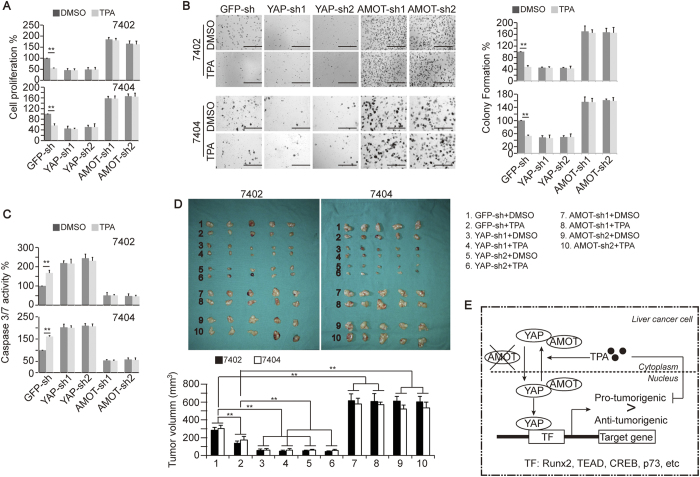
TPA inhibits transformative phenotypes in liver cancer cells via YAP and AMOT. (**A**–**C**) Cell proliferation, colony formation capacity and apoptosis status in control cells and Bel-7402 or Bel-7404 cells with AMOT or YAP knocked down in the presence or absence of DMSO or TPA (final concentration 16 μM), as measured by MTT assay (**A**), soft agar colony formation assay (**B**) and Caspase 3/7 luciferase Glo reagent (**C**), respectively. (**D**) Effects of TPA on the growth of tumors generated by Bel-7402 or Bel-7404 cells under the indicated treatment. Mice were treated with same amount of DMSO or TPA (100 μg/kg) for 20 days after xenografts were visible (starting on day 10 after the initial injection). N = 5/group. A picture of the xenograft is shown in the upper panel, and the data are graphed in the lower panel. (**E**) Possible mechanism underlying how TPA inhibits transformative phenotypes in liver cancer cells via YAP and AMOT. The data are shown as the mean ± SD from three independent experiments (except Fig. 6D). The data from cells “infected with GFP-sh and treated with DMSO” are arbitrarily set to 100%. **p < 0.01. The data from Fig. 6A–C were analyzed using Student’s t-test, and the data from Fig. 6D were analyzed using one-way ANOVA.

Here, we sought to determine whether the TPA-reduced transformative phenotypes in liver cancer cells depend on YAP and AMOT. We found that TPA-reduced cell proliferation and colony formation capacities could be blocked by the knockdown of YAP and AMOT, respectively (Fig. 6A,B). Furthermore, TPA-induced Caspase 3/7 activities could also be abolished by the knockdown of YAP and AMOT, respectively (Fig. 6C). However, TPA still had inhibitory effects on the transformative phenotypes of liver cancer cells, even when the WW domain containing transcription regulator 1 (TAZ), a homolog of YAP, was depleted (Supplementary Fig. S1A-D), thereby excluding the possibility that the effects generated by TPA occur via a TAZ-dependent mechanism.

*In vivo* xenograft experiments also demonstrated that the TPA efficacy with respect to tumor growth inhibition was much reduced when YAP or AMOT was knocked down (Fig. 6D). These results demonstrate that TPA inhibits transformative phenotypes in liver cancer cells, possibly via AMOT and YAP.

## Discussion

Previous studies have demonstrated that TPA enhances cell migration through activating protein kinase C (PKC) in several types of tumor cells^4^,^6^,^7^. As a PKC activator, TPA also inhibits the apoptosis induced by Fas/FasL in human promyelocytic leukemia cells^9^. Although TPA has been reported to be pro-tumorigenic^39^, its exact effects remain controversial. A study from Gong *et al*.^40^ has suggested that TPA exerts opposing roles on cell proliferation, possibly via regulating the Hippo/YAP pathway in a cell type-dependent manner. This may be because TPA can activate different PKC isoforms in different types of cells. For example, TPA affects the nPKC isoform to activate LATS, a natural inhibitor of YAP, in Swiss3T3, MEF and A549 cells, whereas TPA affects the cPKC isoform to suppress LATS in HEK293A cells. Thereby, one drug might cause opposite effects via the same signaling pathway. However, which PKC isoform mediates TPA-induced simultaneous translocation of AMOT and YAP from the nucleus to cytoplasm in liver cancer cells remains unknown and must be investigated in future studies. Unlike the pro-tumorigenic roles of TPA in other types of tumor cells, TPA might act as an anti-tumorigenic agent in the human liver cancer cell line HepG2^8^. Consistent with this possibility, we report here that TPA is able to suppress transformative phenotypes in two human liver cancer cell lines, Bel-7402 and Bel-7404, further suggesting that TPA might be used as an anti-tumor drug in the treatment of liver cancer.

YAP translocation from the nucleus to the cytoplasm might dissolve the YAP-TEAD complex^41^, which promotes cell proliferation and maintains survival programs by inducing the expression of target genes, such as CyclinD1, CyclinE and CTGF^42^,^43^. We found that TPA treatment leads to the separation of YAP from a series of its dependent transcription factors, including TEAD, CREB, Runx2 and p73, whose nuclear localization is not affected by TPA. Notably, these transcription factors play critical roles in the promotion or suppression of liver tumorigenesis^20^,^29^. Because YAP functions as a co-transcription factor, the loss of YAP in the nucleus decreases the transcriptional activities and subsequent target gene expression of its dependent transcription factors. Although YAP-dependent transcription factors are either pro-tumorigenic (for example, TEAD and CREB) or anti-tumorigenic (for example, Runx2 and p73), we believe the major roles of YAP in liver cancer cells are pro-tumorigenic because YAP is highly up-regulated in liver cancer tissues compared to the corresponding adjacent normal liver^19^; YAP knockout restricts liver development^25^; and YAP depletion leads to impaired transformative phenotypes in liver cancer cells^28^. These results might explain why TPA is anti-tumorigenic in liver cancer cells.

AMOT has been identified as a potential component of Hippo signaling, and numerous studies have reported that AMOT inhibits YAP activity^24^,^44^. The interaction between YAP and AMOT has also been well established^45^, and the PPxY motifs of AMOT are essential for interaction with the WW domains within the YAP protein^46^. Our findings have demonstrated that without AMOT, TPA is unable to alter the subcellular localization of YAP or the activities of YAP-dependent transcription factors. Therefore, AMOT is critical for maintaining the efficacy of TPA in treating YAP-dependent liver cancer.

In the present and our previous studies^47^, we found that endogenous AMOT is in the nucleus, whereas exogenous AMOT-HA was observed to be excluded from the nucleus, as most other reports^28^,^45^ have described. Protein synthesis occurs in the cytoplasm and especially on the rough endoplasmic reticulum (ER)^48^. Consequently, exogenous AMOT-HA protein should be abundantly translated on the ER, thus leading to AMOT-HA accumulation in the cytoplasm. It also takes time to transfer AMOT-HA, via an unknown mechanism, to its correct subcellular localization, possibly in the nucleus. We hypothesize that concurrently, the cytoplasmic accumulation of AMOT-HA induces YAP translocation from the nucleus to the cytoplasm, ultimately resulting in its inhibitory effect on YAP. This might explain why AMOT-HA exists mainly in the cytoplasm and might explain the observation that AMOT and YAP appear to bind each other better when they co-localize in the cytoplasm than in the nucleus. The relevant mechanism should be investigated further in the future. Moreover, we have further tested the specificity of the anti-AMOT antibody used in the present study. We found the nuclear staining went away when knocked down AMOT with shRNA, as measured by IF experiment (Fig. S2A). The knockdown efficiencies of AMOT by shRNAs targeting AMOT were also analyzed by WB experiments (Fig. S2B). These results indicated that the nuclear signals that recognized by this anti-AMOT antibody represent genuine endogenous AMOT in the nucleus of Bel-7402 and Bel-7404 cells.

In conclusion, our findings demonstrate that TPA is anti-tumorigenic in liver cancer cells via an AMOT and YAP-dependent mechanism (Fig. 6E). Simultaneously increasing AMOT function might enhance the efficacy of TPA in treating liver cancer.

## Additional Information

**How to cite this article**: Zhu, G. *et al*. 12-O-Tetradecanoylphorbol-13-acetate (TPA) is anti-tumorigenic in liver cancer cells via inhibiting YAP through AMOT. *Sci. Rep.* **7**, 44940; doi: 10.1038/srep44940 (2017).

**Publisher's note:** Springer Nature remains neutral with regard to jurisdictional claims in published maps and institutional affiliations.

## Supplementary Material

Supplementary Figure
