# Supplementary material for: 12-O-Tetradecanoylphorbol-13-acetate (TPA) is anti-tumorigenic in liver cancer cells via inhibiting YAP through AMOT
Source: Sci Rep. 2017 Mar 21;7:44940. doi: 10.1038/srep44940 (PMC5359578; doi:10.1038/srep44940)
Supplement: Supplementary Figure [file srep44940-s1.docx]

12-O-Tetradecanoylphorbol-13-acetate (TPA) is anti-tumorigenic in liver cancer cells via inhibiting YAP through AMOT

Running title: TPA is anti-tumorigenic via YAP

Guoqing Zhu^1^, Yan Chen^1^, Xiao Zhang^1^, Qi Wu^1^, Yinghui Zhao^1^, Yuxin Chen^1^, Fenyong Sun^1^, Yongxia Qiao^2,*^, Jiayi Wang^1,3,**^

1. Department of Clinical Laboratory, Shanghai Tenth People’s Hospital of Tongji University, Shanghai, 200072, China;
2. School of Public Health, Shanghai Jiaotong University School of Medicine, Shanghai, 200025, China;
3. Tongji University Advanced Institute of Translational Medicine, Shanghai, 200092, China;

**Supplementary Figure. S1**


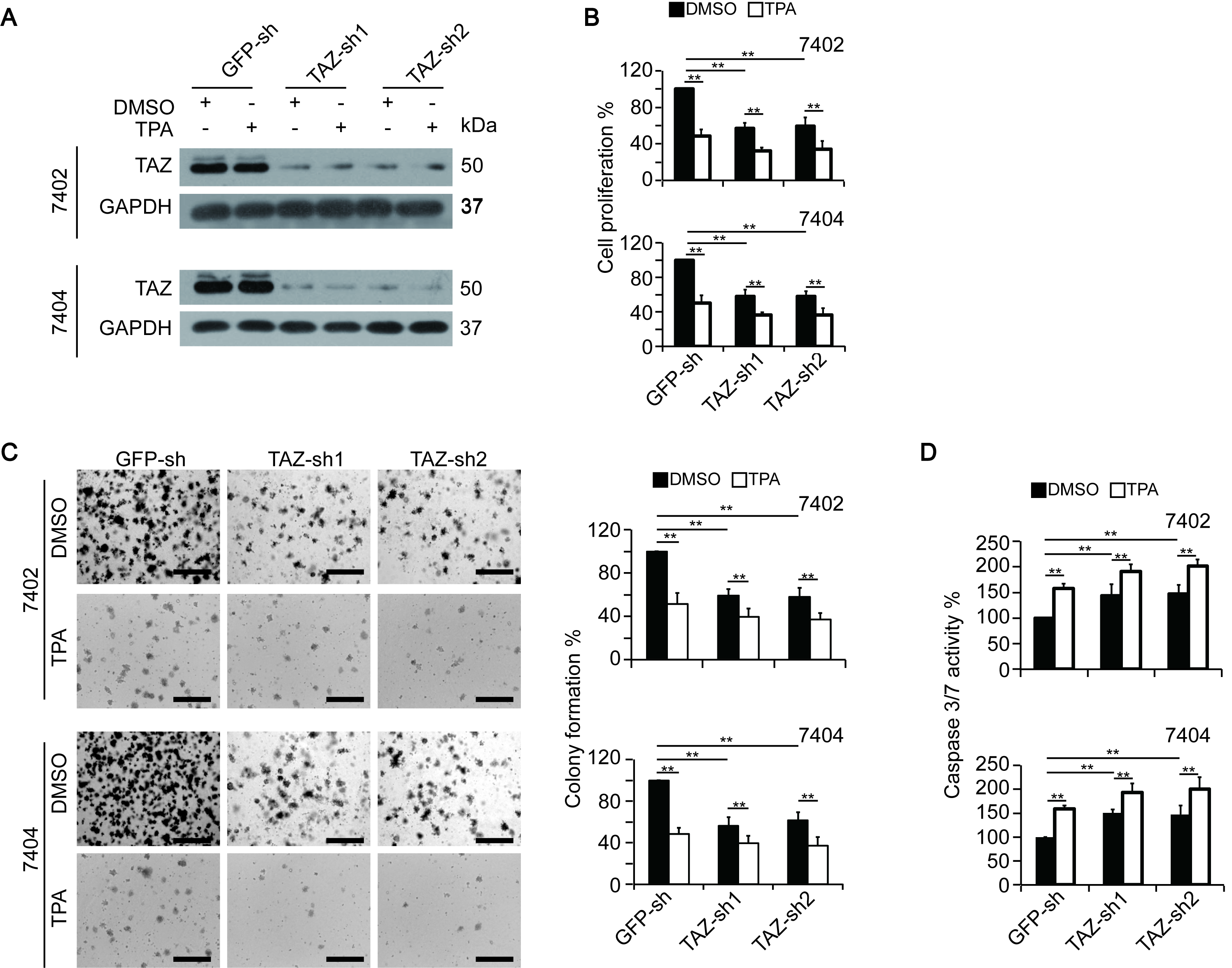


**Figure. S1. TPA-impaired transformative phenotypes is not via TAZ**

(A) Representative Western blots of TAZ under different treatments, as indicated. Control cells, Bel-7402 or Bel-7404 cells with TAZ knocked down (infected with TAZ-sh1 and –sh2, respectively) in the presence or absence of DMSO or TPA (final concentration 16 μM) for 24 h were harvested before Western blotting.

(B-D) Cell proliferation, colony formation capacity and Caspase 3/7 activity were measured by an MTT-base assay (B), soft agar colony formation assay (C) and Caspase 3/7 luciferase Glo reagent (D), respectively, in control cells, Bel-7402 or Bel-7404 cells with TAZ knocked down (infected with TAZ-sh1 and –sh2, respectively) in the presence or absence of DMSO or TPA (final concentration 16 μM) for 24 h. The representative soft agar images from three independent experiments are shown (Fig. S1C, left panel), and the data are also graphed (Fig. S1C, right panel). All the data are shown as mean±SD from three independent experiments. The data from cells “infected with GFP-sh and treated with DMSO” are arbitrarily set to 100 %. **, p<0.01. The data were analyzed using a one-way ANOVA.

**Supplementary Figure. S2**


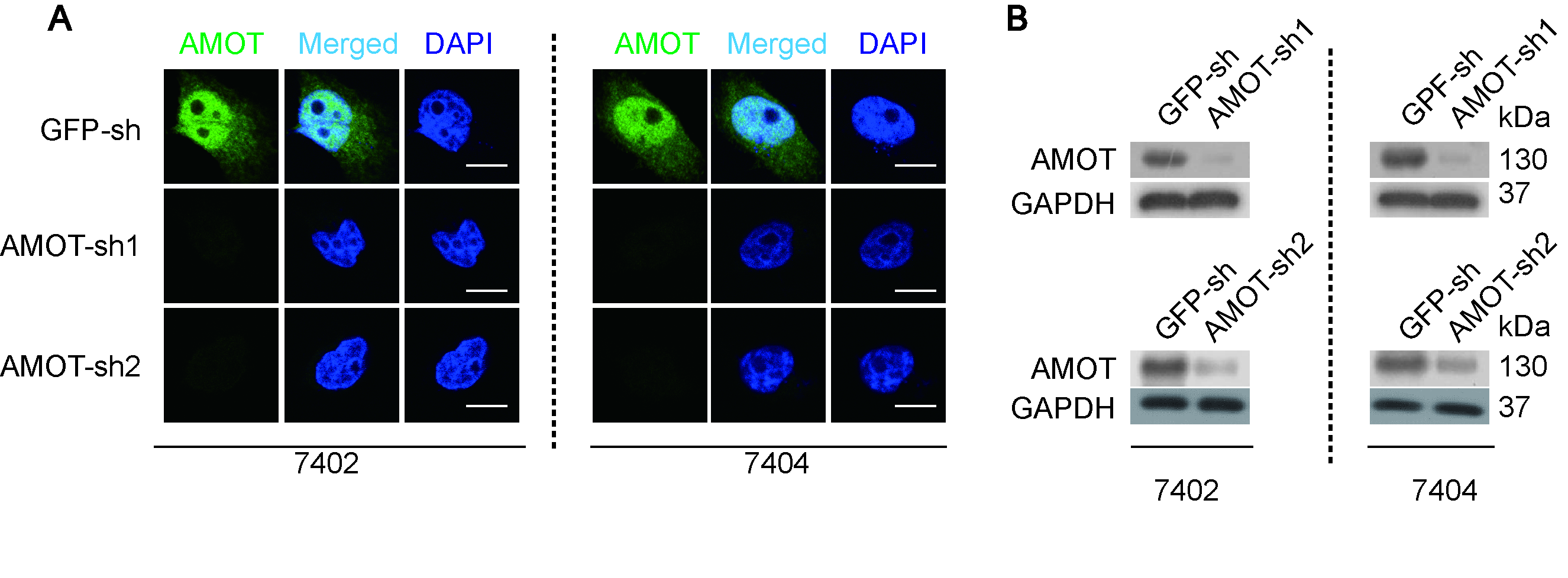


**Figure. S2. Specificity of the anti-AMOT antibody**

(A) Subcellular localization of AMOT in control cells (infected with GFP-sh), and Bel-7402 or Bel-7404 cells with AMOT knocked down (infected with AMOT-sh1 and –sh2, respectively).

(B) Representative Western blots of AMOT in control cells (infected with GFP-sh), and Bel-7402 or Bel-7404 cells with AMOT knocked down (infected with AMOT-sh1 and –sh2, respectively).

All the images are representative ones from three independent experiments.
